# Development and Initial Validation of a Statistical Image Analysis Framework for Quantitative Bone SPECT/CT in Patients with Prostate Cancer

**DOI:** 10.3390/diagnostics16193206

**Published:** 2026-10-02

**Authors:** Kenta Miwa, Noriaki Miyaji, Tensho Yamao, Kosuke Yamashita, Tetsuo Hosoya, Kazunori Kawakami

**Affiliations:** 1 Department of Radiological Sciences, School of Health Sciences, Fukushima Medical University, 10-6 Sakaemachi, Fukushima 960-8516, Japan; miyaji41@fmu.ac.jp (N.M.); tyamao@fmu.ac.jp (T.Y.); 2 Department of Medical Imaging Technology, Faculty of Life Sciences, Kumamoto University, 4-24-1 Kuhonji, Kumamoto 862-0976, Japan; kyamashita@kumamoto-u.ac.jp; 3 PDRadiopharma Inc., 2-14-1 Kyobashi, Tokyo 104-0031, Japan; tetsuo.hosoya@pdradiopharma.com (T.H.); kazunori.kawakami@pdradiopharma.com (K.K.)

**Keywords:** bone SPECT/CT, statistical image analysis, quantitative SPECT, Z-score mapping, normal database, anatomical standardization

## Abstract

**Background/Objectives:** Quantitative bone single-photon emission computed tomography/computed tomography (SPECT/CT) enables standardized uptake value (SUV)-based assessment of skeletal tracer uptake; however, SUV does not directly indicate the degree of deviation from the physiological distribution at the corresponding anatomical location. This study aimed to develop a statistical image analysis software framework for quantitative bone SPECT/CT and perform its initial validation. **Methods:** Eighty-one male patients who underwent quantitative bone SPECT/CT for suspected bone metastases from prostate cancer were retrospectively analyzed, including 49 without and 32 with bone metastases. The developed software integrated CT-based anatomical standardization, construction of a bone-specific normal database (NDB), and voxel-wise Z-score mapping. Thirty successfully standardized subjects without bone metastases were used to construct the NDB. Preliminary clinical validation included 75 reference skeletal regions of interest (ROIs) in the lumbar vertebrae and iliac bones from three independent subjects and 74 metastatic lesions from 19 patients. **Results:** Anatomical standardization was successful in 33/49 subjects without bone metastases and 19/32 patients with bone metastases. NDBs comprising 8, 16, and 30 subjects showed no significant difference in the standard deviation-to-mean ratio (*p* = 0.37). The developed software successfully generated voxel-wise Z-score maps representing deviations from the reference distribution at corresponding skeletal locations. Z-score_max_ showed a strong positive correlation with SUV_max_ in metastatic lesions (ρ = 0.95, *p* < 0.0001), and all 74 metastatic lesions showed Z-score_max_ > 2. **Conclusions:** The developed software enables statistical analysis of quantitative bone SPECT/CT using anatomical standardization, a bone-specific NDB, and voxel-wise Z-score mapping. Z-score mapping provides quantitative information relative to the location-specific reference distribution and might complement conventional SUV-based assessment. Further validation using larger and independent cohorts is required to determine its clinical value.

## 1. Introduction

Quantitative single-photon emission computed tomography/computed tomography (SPECT/CT) has expanded the role of bone scintigraphy from visual interpretation to quantitative assessment of radiotracer uptake. Advances in attenuation and scatter correction, image reconstruction, and system calibration have enabled the measurement of standardized uptake values (SUVs) in bone SPECT/CT [1,2,3]. SUV has been investigated as a quantitative imaging biomarker for the assessment of skeletal tracer uptake and bone metastases [4,5,6,7,8].

The EANM practice guideline for quantitative SPECT/CT identifies bone disease as a leading clinical application of quantitative SPECT/CT and highlights that validated normal ranges might improve diagnostic confidence and facilitate automated assessment of disease burden [9]. The guideline also emphasizes the importance of harmonized acquisition and reconstruction when applying absolute quantitative thresholds [9]. However, SUV-based evaluation of bone SPECT/CT has several challenges. Physiological tracer uptake in normal bone varies among individuals and skeletal regions. Recent studies have demonstrated inter-individual and regional variability in vertebral and pelvic SUV, with quantitative values also associated with patient characteristics such as sex, body size, and CT values [4,10]. These physiological variations complicate the interpretation of an individual SUV without an appropriate reference for the corresponding skeletal location. Although SUV can differentiate bone metastases from reference bone or degenerative changes, the distributions of SUV can overlap, and the optimal cutoff values depend on the populations and skeletal abnormalities being evaluated [5,6,7,8]. Therefore, an approach that evaluates tracer uptake according to its deviation from the reference distribution at each skeletal location could provide complementary information to conventional SUV-based quantification.

Statistical image analysis provides such an approach by comparing an individual image with a reference population after anatomical standardization. In brain SPECT imaging, statistical image analysis using a normal database (NDB) and Z-score-based assessment has been applied for objective evaluation of cerebral perfusion abnormalities [11,12,13,14,15]. The easy Z-score Imaging System (eZIS) enables anatomically standardized SPECT images to be compared with an NDB and visualizes the degree of deviation from the reference tracer distribution as Z-score maps [11,12,13,14]. A similar concept has also been applied to torso ^18^F-fluorodeoxyglucose (FDG) PET, demonstrating that anatomical standardization and voxel-wise comparison with a normal model can extend statistical image analysis beyond brain imaging [16,17].

Application of this concept to quantitative bone SPECT/CT could provide a new approach to skeletal image analysis. Anatomical standardization allows the same skeletal locations to be compared across individuals, while a bone-specific NDB provides the mean and variability of physiological tracer uptake at each voxel. Consequently, Z-score mapping can quantify the degree to which tracer uptake in an individual patient deviates from the reference distribution at the corresponding skeletal location. However, a statistical image analysis framework integrating anatomical standardization, a bone-specific NDB, and voxel-wise Z-score mapping has not been developed for quantitative bone SPECT/CT.

Therefore, a statistical image analysis framework for quantitative bone SPECT/CT incorporating CT-based anatomical standardization, bone-specific NDB construction, and voxel-wise Z-score mapping was developed. The present study aimed to perform an initial technical and clinical validation of the framework for quantitative assessment of skeletal abnormalities.

## 2. Materials and Methods

### 2.1. Study Population and Bone SPECT/CT Acquisition

This retrospective study was conducted in accordance with the Declaration of Helsinki and was approved by the institutional review board (approval No. 16-Io-221). The requirement for written informed consent was waived because of the retrospective study design. This study included 81 male patients who underwent quantitative bone SPECT/CT for suspected bone metastases from prostate cancer. Forty-nine patients without bone metastases were classified as the non-metastatic group, and 32 patients with bone metastases were classified as the metastatic group. The mean age, height, and body weight of the study population were 70.07 ± 8.19 years (range, 47–92 years), 166.43 ± 5.27 cm (range, 157–180 cm), and 66.69 ± 9.91 kg (range, 42–94 kg), respectively.

Bone SPECT/CT was performed using a Symbia Intevo system (Siemens Healthineers, Erlangen, Germany). Patients received 991.09 ± 88.73 MBq (range, 785.4–1097.4 MBq) of ^99m^Tc-methylene diphosphonate (^99m^Tc-MDP), and SPECT/CT imaging was performed 2–5 h after administration. SPECT data were acquired using a low-energy high-resolution (LEHR) collimator with 60 views at 3° steps and an acquisition time of 8–10 s per view. Computed tomography (CT) images were acquired at 130 kV with a tube current of 162.08 ± 39.72 mA (range, 24–287 mA) and an exposure time of 600 ms. The imaging range extended from the abdomen to the pelvis. CT-based attenuation correction and dual-energy-window scatter correction were applied. SPECT images were reconstructed using Flash 3D with a matrix size of 256 × 256, 30 iterations, 1 subset, and a Gaussian filter with a full width at half maximum (FWHM) of 6 mm. The reconstructed pixel size was 2.4 mm.

### 2.2. Development of the Statistical Image Analysis Software

Dedicated software for statistical image analysis of quantitative bone SPECT/CT was developed in collaboration with PDRadiopharma Inc. (Tokyo, Japan). The software was designed to generate voxel-wise Z-score maps using anatomical standardization, a bone-specific NDB, and voxel-wise statistical comparison with the NDB.

Projection data and corresponding CT images acquired by SPECT/CT were used as input data. The software automatically performed attenuation correction, scatter correction, image reconstruction, anatomical standardization, count normalization, smoothing, and masking to exclude non-skeletal structures, including the kidneys and soft tissues. The main outputs included preprocessed CT images, SPECT images before and after anatomical standardization, count-normalized standardized SPECT images, projection images after scatter correction when applicable, CT-based reference images for calculating anatomical standardization parameters, SUV images, Z-score images, and text files containing quantitative volume-of-interest (VOI) values.

The CT template used for anatomical standardization was constructed from 10 subjects without bone metastases. For all CT images, the patient table and soft tissues were removed, and the matrix size was converted to 256 × 256. One representative subject was randomly selected from the 10 subjects and used as the initial reference. The CT images of the remaining subjects were anatomically standardized to the reference CT image using Statistical Parametric Mapping 8 (SPM8; Wellcome Trust Centre for Neuroimaging, University College London, London, UK). An average CT image was then generated from the anatomically standardized CT images. All original CT images were subsequently anatomically standardized to this average CT image, and a second average image was generated and used as the final CT template.

**Figure 1:**
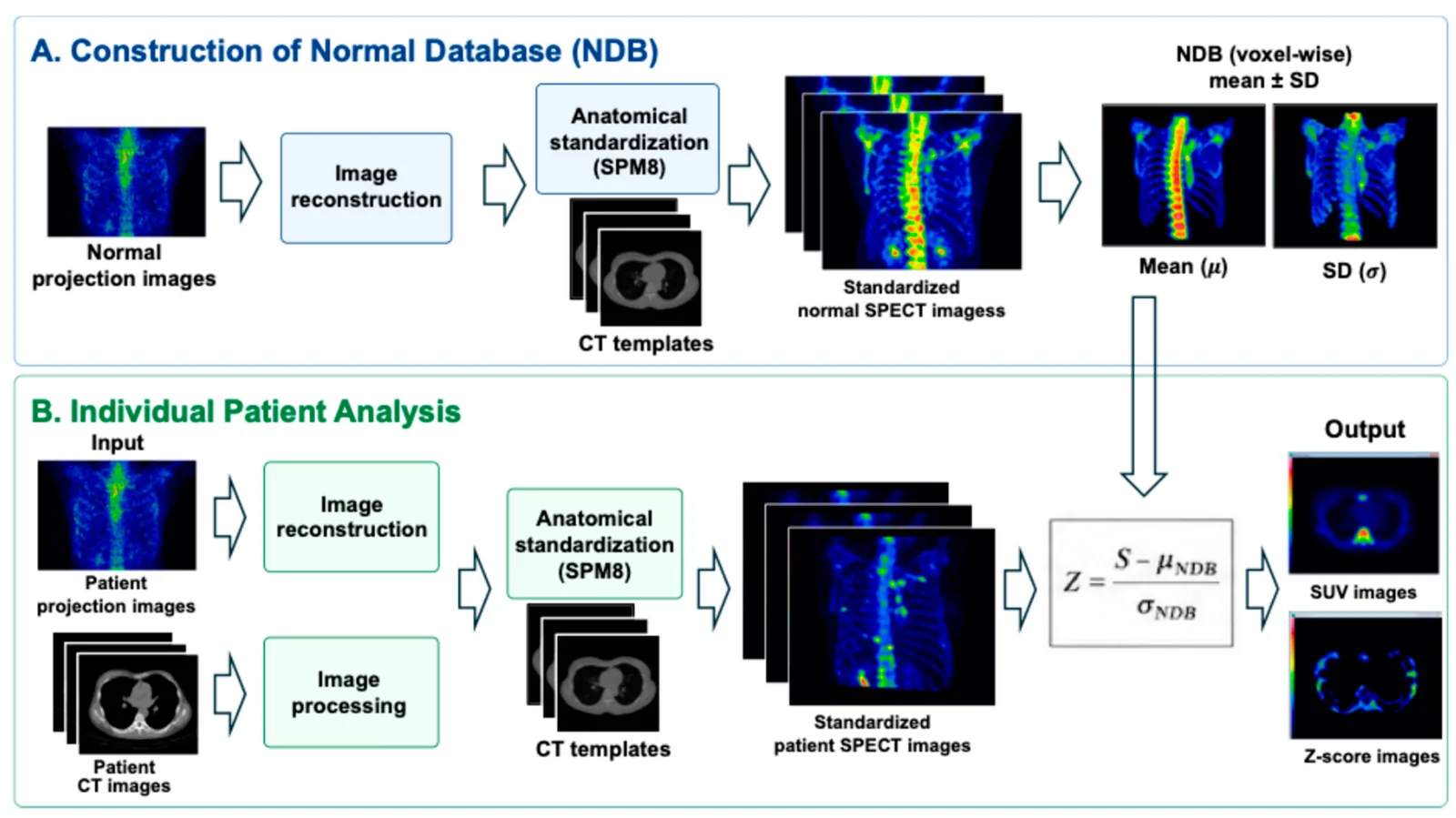
Workflow of the statistical image analysis framework. (**A**) Construction of the bone-specific normal database (NDB) using anatomically standardized SPECT images from subjects without bone metastases. (**B**) Individual patient analysis, including image reconstruction, CT-based anatomical standardization, and voxel-wise comparison with the NDB to generate Z-score and SUV images.

Figure 1 shows the overall workflow of the developed software. First, the projection data were reconstructed to generate quantitative SPECT images. CT-based anatomical standardization was then performed using SPM8 by registering the individual CT image to the CT template and calculating spatial transformation parameters on a voxel-by-voxel basis. The transformation parameters derived from the CT image were applied to the corresponding reconstructed SPECT image to generate an anatomically standardized SPECT image. Standardized SPECT images from subjects without bone metastases were then used to generate voxel-wise mean and standard deviation (SD) images, which constituted the bone-specific NDB. Finally, an individual standardized SPECT image was compared voxel by voxel with the NDB to generate a Z-score map.

The accuracy of anatomical standardization was visually evaluated by superimposing the standardized SPECT image on the CT template and assessing the spatial alignment of the skeletal structures. Cases with visually acceptable alignment between the standardized SPECT image and the corresponding skeletal structures of the CT template were classified as successful anatomical standardization.

### 2.3. Construction and Evaluation of the Normal Database

Anatomical standardization was successful in 33 subjects without bone metastases. Of these, 30 subjects were used to construct the bone-specific NDB, while the remaining three subjects were independently used for preliminary clinical validation and were not included in NDB construction.

For each voxel, the mean and SD of the normalized SPECT counts across the 30 subjects were calculated to generate voxel-wise mean and SD images. The Z-score for each voxel of an individual standardized SPECT image was calculated as follows:

$$Z-\mathrm{score}=\frac{S-\mu_{\mathrm{NDB}}}{\sigma_{\mathrm{NDB}}}$$

where *S* represents the voxel value of the individual standardized SPECT image, and *μ_NDB_* and *σ_NDB_* represent the mean and SD, respectively, of the corresponding voxel in the NDB. The resulting Z-score map represents the degree of deviation in tracer uptake from the normal reference distribution at each skeletal location.

To preliminarily evaluate the influence of NDB size, NDB-8 and NDB-16 were constructed as randomly selected subsets of the 30 subjects constituting NDB-30; thus, these databases were not independent subject groups. The variability of each NDB was evaluated using the voxel-wise mean and SD images. Values were measured at five predefined locations, and SD/mean was calculated at each location. The SD/mean values were compared among NDB-8, NDB-16, and NDB-30. NDB-30 was used for subsequent Z-score analysis.

### 2.4. Preliminary Clinical Validation

Preliminary clinical validation was performed using SUV and Z-score images generated with NDB-30. Three subjects without bone metastases and 19 patients with bone metastases who showed successful anatomical standardization were included in the region-of-interest (ROI) analysis.

In the non-metastatic group, reference ROIs were placed in the lumbar vertebrae and iliac bones of three subjects, resulting in a total of 75 ROIs. In the metastatic group, 74 metastatic lesions from 19 patients were evaluated.

All candidate reference skeletal sites were reviewed by an experienced nuclear medicine physician based on the SPECT/CT findings and available clinical information. Reference ROIs were placed in skeletal sites without apparent focal abnormalities on SPECT/CT. Bone metastases were identified by an experienced nuclear medicine physician based on the SPECT/CT findings and available clinical information. Circular ROIs were placed within the bone without extending beyond the osseous margins. For metastatic lesions, ROIs were placed at the center of each lesion. When multiple metastatic lesions were present in the same patient, each lesion was evaluated separately using an individual ROI. SUV_max_ and Z-score_max_ were defined as the maximum SUV and Z-score within each ROI, respectively. The relationship between SUV_max_ and Z-score_max_ was evaluated separately for reference skeletal sites and metastatic lesions.

### 2.5. Statistical Analysis

Continuous variables are presented as mean ± SD and range where appropriate. Differences in SD/mean among NDB-8, NDB-16, and NDB-30 were evaluated using the Friedman test. The relationships between SUV_max_ and Z-score_max_ were assessed using Spearman’s rank correlation coefficient. For the SUV_max_–Z-score_max_ correlation analysis, the individual ROI or metastatic lesion was used as the unit of analysis. A *p* value of <0.05 was considered statistically significant. Statistical analyses were performed using GraphPad Prism 7, version 7.0c (GraphPad Software, La Jolla, CA, USA).

### 2.6. Use of Generative Artificial Intelligence

During the preparation and revision of this manuscript, ChatGPT (OpenAI, GPT-5.6) was used to assist with drafting and refining manuscript text, including grammar and language editing. The tool was not used for study design, data collection, data generation, statistical analysis, or interpretation of the study results. All AI-assisted output was reviewed and edited by the authors, who take full responsibility for the content of this publication.

## 3. Results

### 3.1. Software Implementation and Anatomical Standardization

**Figure 2:**
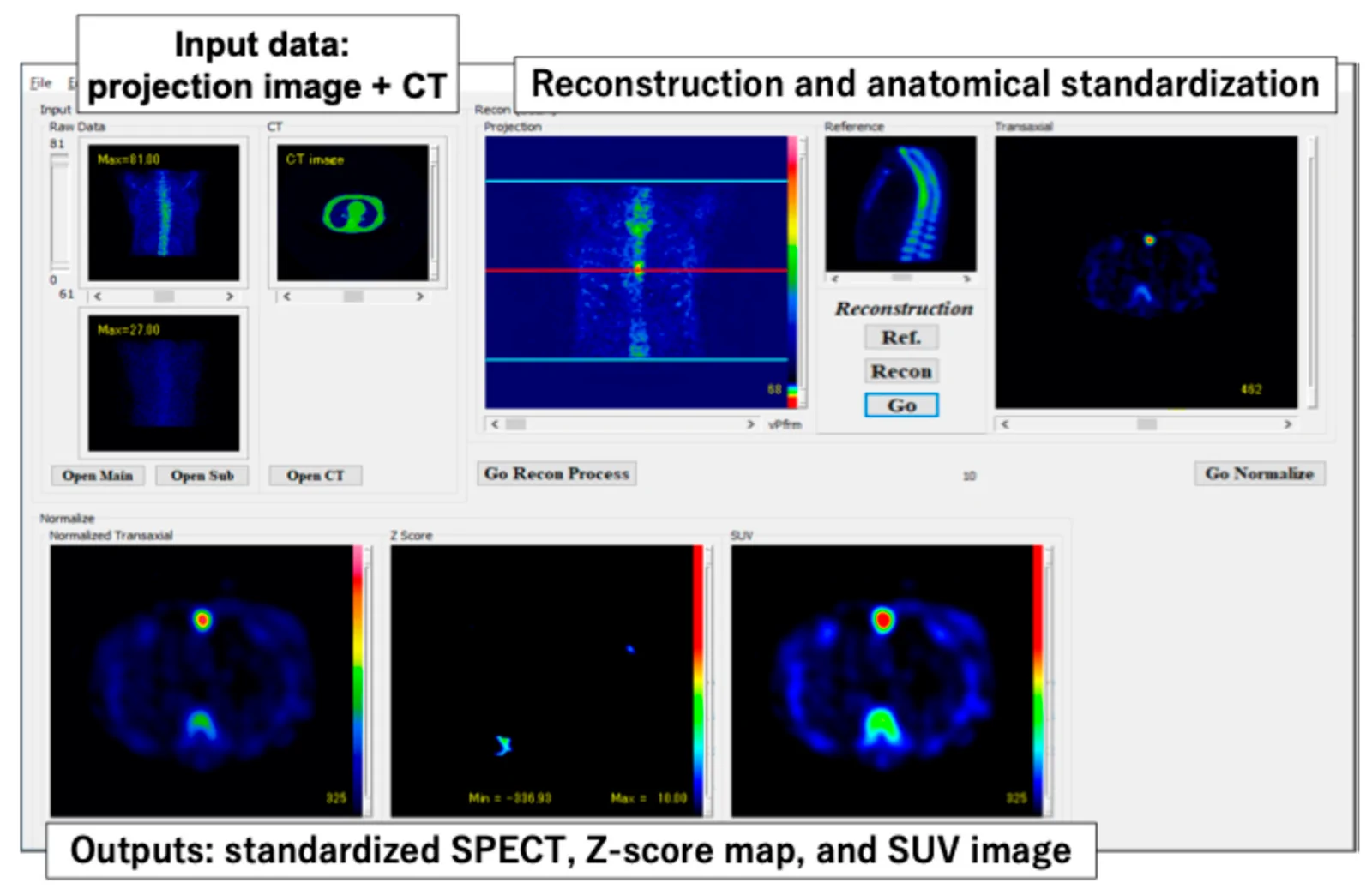
Main window of the developed statistical image analysis software (eZIS BONE). The software automatically performs attenuation correction, scatter correction, image reconstruction, anatomical standardization, count normalization, smoothing, and masking to exclude non-skeletal structures, including the kidneys and soft tissues. The outputs include preprocessed CT images, SPECT images before and after anatomical standardization, count-normalized standardized SPECT images, scatter-corrected projection images, CT-based reference images for anatomical standardization, SUV images, Z-score images, and text files containing quantitative VOI values.

The developed software successfully processed the projection and CT data and generated anatomically standardized SPECT images, SUV images, and voxel-wise Z-score maps. Figure 2 shows representative images generated using the software.

Anatomical standardization was evaluated in all 81 patients. Successful anatomical standardization was achieved in 33 of 49 subjects without bone metastases (67.3%) and 19 of 32 patients with bone metastases (59.4%). Anatomical standardization was unsuccessful in 16 (32.7%) and 13 (40.6%) subjects, respectively.

**Figure 3:**
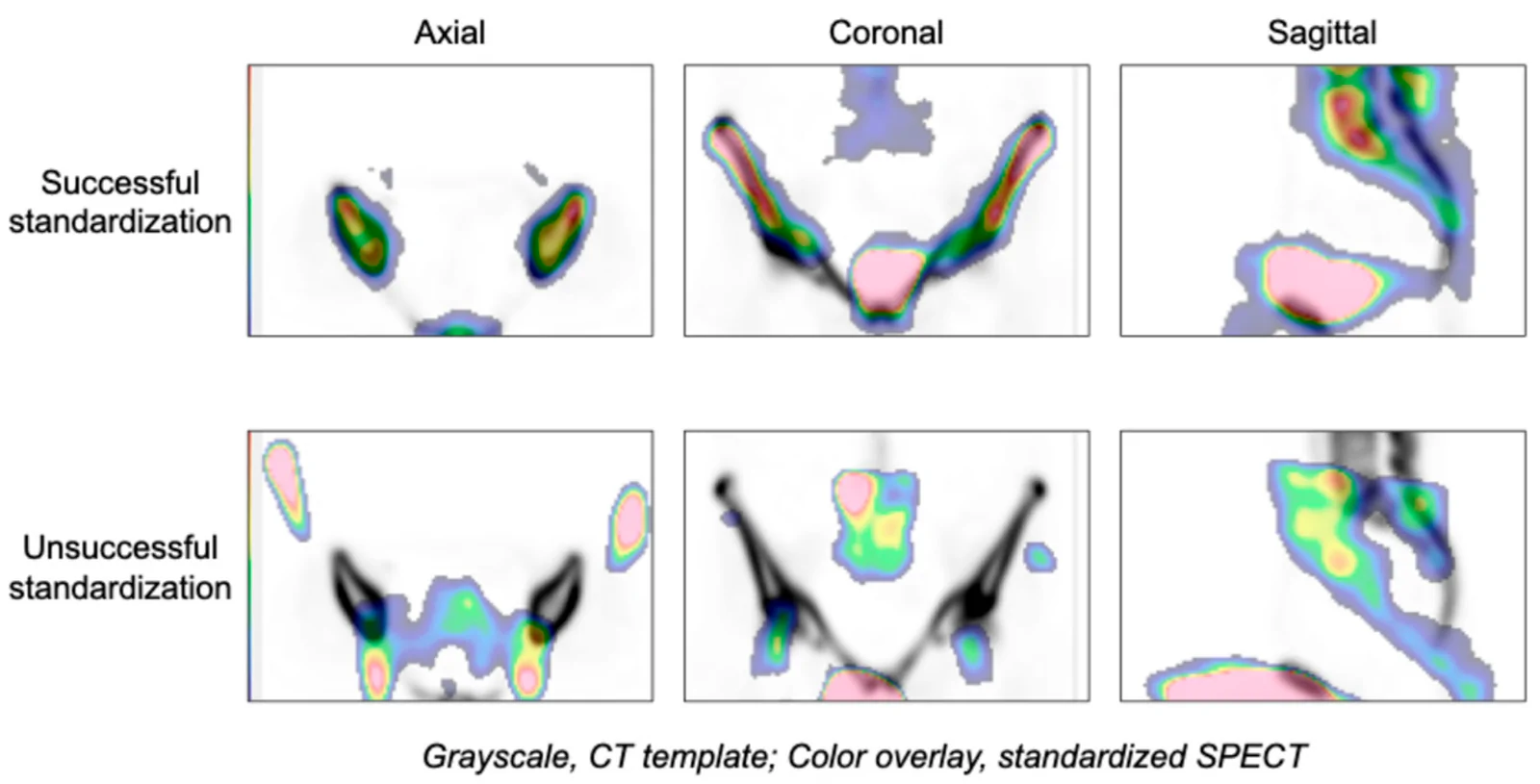
Representative examples of successful and unsuccessful anatomical standardization. The unsuccessful case shows anterior displacement of the spine and misalignment of the pelvic region.

Figure 3 shows representative cases with successful and unsuccessful anatomical standardization. In the successful case, the standardized SPECT image was generally aligned with the CT template. In the unsuccessful case, anterior displacement of the spine and misalignment of the pelvic region were observed.

### 3.2. Normal Database Construction and Evaluation

Bone-specific NDBs were successfully generated from anatomically standardized SPECT images. The median SD/mean values were 17.2% for NDB-8, 13.8% for NDB-16, and 14.9% for NDB-30. No significant difference in SD/mean was observed among the three NDBs (*p* = 0.37).

### 3.3. Preliminary Clinical Validation

Preliminary clinical validation included 75 reference skeletal ROIs from the lumbar vertebrae and iliac bones of three subjects without bone metastases and 74 metastatic lesions from 19 patients. The mean SUV_max_ was 12.84 ± 3.16 in reference skeletal sites and 19.97 ± 19.87 in metastatic lesions, with median values of 11.98 and 15.15, respectively. The mean Z-score_max_ was 2.31 ± 0.94 in reference skeletal sites and 10.69 ± 16.69 in metastatic lesions, with median values of 2.17 and 6.32, respectively.

**Figure 4:**
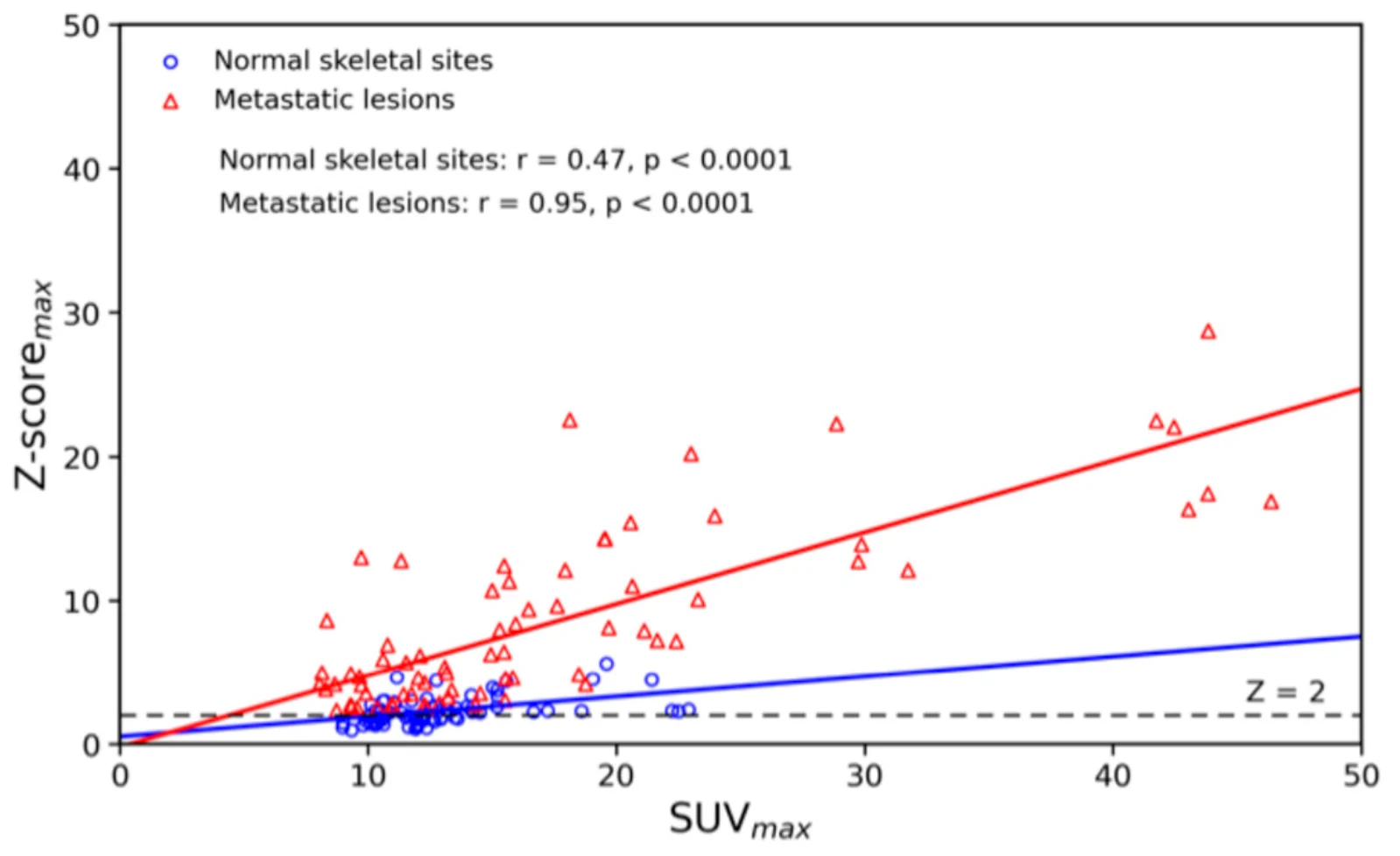
Relationship between SUV_max_ and Z-score_max_ in reference skeletal sites and metastatic lesions. The dashed horizontal line indicates Z-score_max_ = 2. Spearman correlation analysis demonstrated a moderate positive correlation in reference skeletal sites (ρ = 0.47) and a strong positive correlation in metastatic lesions (ρ = 0.95). Both correlations were statistically significant (*p* < 0.0001). The graph shows a magnified range for visualization; all data, including values outside the plotted axes, were included in the correlation analysis.

All 74 metastatic lesions showed a Z-score_max_ > 2, whereas Z-score_max_ > 2 was also observed in some reference skeletal sites. SUV_max_ and Z-score_max_ showed a strong positive correlation in metastatic lesions (ρ = 0.95, *p* < 0.0001) and a moderate positive correlation in reference skeletal sites (ρ = 0.47, *p* < 0.0001). Table 1 summarizes the quantitative parameters and correlation coefficients. Figure 4 shows the relationship between SUV_max_ and Z-score_max_ in reference skeletal sites and metastatic lesions.

## 4. Discussion

The present study developed a statistical image analysis software framework for quantitative bone SPECT/CT and demonstrated the feasibility of CT-based anatomical standardization, bone-specific NDB construction, and voxel-wise Z-score mapping. The developed software successfully generated Z-score maps from clinical bone SPECT/CT data, and Z-score_max_ showed a strong positive correlation with SUV_max_ in metastatic lesions. These findings indicate that the developed framework enables quantitative assessment of skeletal tracer uptake as a deviation from the reference distribution at the corresponding anatomical location, providing information complementary to conventional SUV-based assessment.

A major advantage of the developed software is that it provides statistical information on skeletal tracer uptake based on anatomically corresponding reference data. Quantitative bone SPECT/CT enables absolute quantification using SUV, and previous studies have demonstrated its clinical utility for evaluating bone metastases [1,5,6,7,8]. However, physiological skeletal uptake shows considerable inter-individual variability and varies according to skeletal location and patient characteristics [4,10]. Hou et al. quantified physiological ^99m^Tc-MDP uptake in the normal spine and pelvis and demonstrated regional variability in quantitative values [4], while a larger study of 2416 normal vertebrae demonstrated associations of SUV with sex, body size, and CT values [10]. The developed software addresses these variations by comparing individual uptake with the mean and SD of the NDB at each corresponding skeletal location. Therefore, the Z-score represents not only the magnitude of tracer uptake but also how much the uptake deviates from the expected reference distribution.

The clinical utility of eZIS and related Z-score-based statistical image analysis has been demonstrated in brain SPECT using anatomical standardization and normal databases [11,12,13,14], and the influence of reconstruction and scanner characteristics on the performance of SPECT normal databases has also been investigated [15]. A similar approach was applied to torso FDG PET using anatomical standardization and a voxel-wise normal model [16,17]. The present study extends this concept to quantitative bone SPECT/CT and provides a dedicated software framework for voxel-wise assessment of skeletal tracer uptake relative to a bone-specific reference distribution. Unlike conventional ROI-based SUV analysis, the developed software generates a Z-score map across the analyzed skeletal region, allowing the spatial distribution and extent of deviations from reference skeletal uptake to be visualized.

The strong correlation between SUV_max_ and Z-score_max_ in metastatic lesions should be interpreted in the context of their different meanings. All 74 metastatic lesions showed Z-score_max_ values greater than 2, and the median Z-score_max_ was 6.32, compared with 2.17 in reference skeletal sites. SUV represents the magnitude of tracer uptake, whereas the Z-score represents the deviation from the location-specific reference distribution. Thus, Z-score analysis is not intended to replace SUV but to provide complementary statistical information by expressing uptake relative to the NDB. The EANM practice guideline for quantitative SPECT/CT also highlights the potential value of validated normal ranges for improving diagnostic confidence and facilitating automated assessment of disease burden [9]. Whether this complementary information improves diagnostic performance requires further validation. Because Z-score analysis accounts for the location-specific mean and variability of reference skeletal uptake, it might reduce the influence of physiological regional variations that complicate interpretation of absolute SUV. However, its ability to differentiate metastatic lesions from degenerative skeletal changes was not specifically evaluated in the present study.

The bone-specific NDB and CT-based anatomical standardization are essential for voxel-wise Z-score analysis. The median SD/mean values were 17.2%, 13.8%, and 14.9% for NDB-8, NDB-16, and NDB-30, respectively, with no significant difference among the three databases. The NDB provides a reference distribution for evaluating individual skeletal uptake at anatomically corresponding locations, while CT-based anatomical standardization establishes the spatial correspondence required for voxel-wise comparison. CT-based anatomical standardization has also been used for statistical analysis of torso FDG-PET/CT [16,17], supporting the feasibility of using anatomical CT information to construct voxel-wise reference models. Further expansion of the NDB and refinement of anatomical standardization might improve the precision and robustness of Z-score analysis.

Z-score_max_ > 2 was also observed in some reference skeletal sites. Therefore, the present results do not establish a Z-score_max_ threshold for the diagnosis of bone metastasis. Rather, they demonstrate that the developed software can quantify the degree of deviation in skeletal tracer uptake from a reference distribution. The diagnostic potential of Z-score analysis might not be limited to its maximum value. The spatial extent and distribution of elevated Z-scores might provide additional information on abnormal skeletal uptake and should be evaluated in larger independent cohorts.

This study has several limitations. First, the bone-specific NDB was constructed from a limited number of subjects acquired using a single SPECT/CT system and imaging protocol. Further validation using larger datasets is required to establish the robustness and generalizability of the proposed framework. Second, all subjects were male patients with prostate cancer, and detailed information regarding hormonal treatment was not available for all patients in the retrospective dataset. Therefore, further validation is required in other populations, and the potential influence of hormonal treatment on bone tracer uptake could not be reliably evaluated. Third, exact injection times were not available for all patients; therefore, the influence of individual uptake-time variability could not be reliably evaluated. Fourth, the quantitative validation using reference ROIs was performed in the lumbar vertebrae and iliac bones; therefore, further validation is required for other skeletal regions. Finally, anatomical standardization was not successful in all subjects and was evaluated visually. Because accurate anatomical correspondence is essential for voxel-wise comparison with the NDB, further optimization of the registration parameters is required to improve the accuracy and robustness of anatomical standardization. A higher and more consistent success rate of anatomical standardization will be important for the clinical implementation of the developed software. Further studies using larger NDBs, optimized anatomical standardization, and independent patient cohorts are needed to evaluate the diagnostic performance and broader clinical applicability of the developed software.

## 5. Conclusions

In conclusion, the proposed statistical image analysis framework enabled voxel-wise Z-score mapping of quantitative bone SPECT/CT relative to an NDB. The preliminary results demonstrated the feasibility of this approach in patients with prostate cancer, with clinical quantitative evaluation performed in the lumbar vertebrae and iliac bones. Z-score analysis provides statistical information on tracer uptake relative to the location-specific reference distribution and might complement conventional SUV-based assessment. Further studies using larger and independent cohorts are required to determine its additional clinical value.

## Figures and Tables

**Table 1:** Quantitative parameters and correlations between SUV_max_ and Z-score_max_.

Parameter	Reference Skeletal Sites	Metastatic Lesions
Number of ROIs/lesions	75	74
SUV_max_, mean ± SD	12.84 ± 3.16	19.97 ± 19.87
Z-score_max_, mean ± SD	2.31 ± 0.94	10.69 ± 16.69
Spearman’s ρ (SUV_max_ vs. Z-score_max_)	0.47	0.95
*p* value	<0.0001	<0.0001

Note: ROI, region of interest; SD, standard deviation. Correlations were assessed using Spearman’s rank correlation coefficient.

## Data Availability

The data supporting the findings of this study are available from the corresponding author upon reasonable request. The data are not publicly available due to privacy and ethical restrictions.

## Abbreviations

The following abbreviations are used in this manuscript:

SPECT	Single-photon emission computed tomography
CT	Computed tomography
SUV	Standardized uptake value
NDB	Normal database
ROI	Region of interest
SD	Standard deviation
LEHR	Low-energy high-resolution
^99m^Tc-MDP	Technetium-99m methylene diphosphonate
eZIS	Easy Z-score imaging system
FDG	Fluorodeoxyglucose
PET	Positron emission tomography

## References

[1] Zivanovic J., Zhang-Yin J. (2026). Quantification in bone SPECT/CT. Front. Nucl. Med..

[2] Miyaji N., Miwa K., Tokiwa A., Ichikawa H., Terauchi T., Koizumi M., Onoguchi M. (2020). Phantom and clinical evaluation of bone SPECT/CT image reconstruction with xSPECT algorithm. EJNMMI Res..

[3] Miwa K., Nemoto R., Masuko H., Yamao T., Kobayashi R., Miyaji N., Inoue K., Onodera H. (2022). Evaluation of quantitative accuracy among different scatter corrections for quantitative bone SPECT/CT imaging. PLoS ONE.

[4] Hou X., He Y., Liu G., Chen S., Shi H. (2024). SPECT/CT imaging: Quantifying ^99m^Tc-MDP concentration in the spine and pelvis. Ann. Nucl. Med..

[5] Kuji I., Yamane T., Seto A., Yasumizu Y., Shirotake S., Oyama M. (2017). Skeletal standardized uptake values obtained by quantitative SPECT/CT as an osteoblastic biomarker for the discrimination of active bone metastasis in prostate cancer. Eur. J. Hybrid Imaging.

[6] Ikeda T., Kitajima K., Tsuchitani T., Takahashi Y., Hama Y., Kotura N. (2022). Effectiveness of quantitative bone SPECT/CT for bone metastasis diagnosis. Hell. J. Nucl. Med..

[7] Tabotta F., Jreige M., Schaefer N., Becce F., Prior J.O., Nicod Lalonde M. (2019). Quantitative bone SPECT/CT: High specificity for identification of prostate cancer bone metastases. BMC Musculoskelet. Disord..

[8] Vija A.H., Bartenstein P.A., Froelich J.W., Kuwert T., Macapinlac H., Daignault C.P., Gowda N., Hadjiev O., Hephzibah J., Huang P. (2019). ROC study and SUV threshold using quantitative multi-modal SPECT for bone imaging. Eur. J. Hybrid Imaging.

[9] Dickson J.C., Armstrong I.S., Minguez Gabiña P., Denis-Bacelar A.M., Krizsan A.K., Gear J.M., Van den Wyngaert T., de Geus-Oei L.F., Herrmann K. (2023). EANM practice guideline for quantitative SPECT-CT. Eur. J. Nucl. Med. Mol. Imaging.

[10] Qi N., Meng Q., You Z., Chen H., Shou Y., Zhao J. (2021). Standardized uptake values of ^99m^Tc-MDP in normal vertebrae assessed using quantitative SPECT/CT for differentiation diagnosis of benign and malignant bone lesions. BMC Med. Imaging.

[11] Kanetaka H., Shimizu S., Inagawa Y., Hirose D., Takenoshita N., Sakurai H., Hanyu H. (2020). Differentiating Mild Cognitive Impairment, Alzheimer’s Disease, and Dementia With Lewy Bodies Using Cingulate Island Sign on Perfusion IMP-SPECT. Front. Neurol..

[12] Wang S., Qi Y., Jiang Y., Chi X., Huang K., Ruan C., Yang X., Li G. (2021). Analysis of brain perfusion single-photon emission tomography images using an easy Z-score imaging system for early diagnosis of Alzheimer’s disease. J. South. Med. Univ..

[13] Yamamoto S., Yoshida N., Sakurai N., Okada Y., Satoh M., Takeshita K., Nakai M., Abe K., Yoshimura M., Saito K. (2025). Clinical Utility of eZIS in Cerebral Blood Flow SPECT. Diagnostics.

[14] Hayashi H., Kobayashi R., Kawakatsu S., Morioka D., Otani K. (2020). Utility of Easy Z-Score Imaging System-Assisted SPECT in Detecting Onset Age-Dependent Decreases in Cerebral Blood Flow in the Posterior Cingulate Cortex, Precuneus, and Parietal Lobe in Alzheimer’s Disease with Amyloid Accumulation. Dement. Geriatr. Cogn. Disord. Extra.

[15] Dickson J.C., Tossici-Bolt L., Sera T., Booij J., Ziebell M., Morbelli S., Assenbaum-Nan S., Vander Borght T., Pagani M., Kapucu O.L. (2017). The impact of reconstruction and scanner characterisation on the diagnostic capability of a normal database for [123I]FP-CIT SPECT imaging. EJNMMI Res..

[16] Hara T., Kobayashi T., Ito S., Zhou X., Katafuchi T., Fujita H. (2015). Quantitative Analysis of Torso FDG-PET Scans by Using Anatomical Standardization of Normal Cases from Thorough Physical Examinations. PLoS ONE.

[17] Takeda K., Hara T., Zhou X., Katafuchi T., Kato M., Ito S., Ishihara K., Kumita S., Fujita H. (2017). Normal model construction for statistical image analysis of torso FDG-PET images based on anatomical standardization by CT images from FDG-PET/CT devices. Int. J. Comput. Assist. Radiol. Surg..

